# “The community’s memory is timeless”: exploring cultural and community context to inform the public health response to the overdose crisis in black communities

**DOI:** 10.1186/s12954-026-01457-3

**Published:** 2026-04-29

**Authors:** Jasmine Barnes, M. Holliday Davis, Kathryn Gallagher, Kathryn Morris, Nicole O’Donnell, Gilly Gehri, Jeanmarie Perrone, Margaret Lowenstein

**Affiliations:** 1 Center for Addiction Medicine and Policy, University of Pennsylvania, 3801 Filbert Street, Suite 206, Philadelphia, PA 19104 USA; 2 Division of General Internal Medicine, Perelman School of Medicine, University of Pennsylvania, Philadelphia, PA 19104 USA; 3 School of Nursing, University of Pennsylvania, 418 Curie Boulevard, Philadelphia, PA 19104 USA; 4 Division of Medical Toxicology and Addiction Medicine Initiatives, Department of Emergency Medicine, University of Pennsylvania, Philadelphia, PA USA; 5 Leonard Davis Institute for Health Economics, University of Pennsylvania, 3641 Locust Walk, Philadelphia, PA 19104 USA

**Keywords:** Structural racism, Overdose, Harm reduction, Racial disparities, Public health, Substance use disorder (SUD)

## Abstract

**Background:**

Substance use treatment and harm reduction strategies are vital tools in addressing the overdose crisis, however, effectiveness depends on access and uptake. Little is known about perceptions of harm reduction and substance use treatment efforts among people who use drugs (PWUD) in minoritized communities and how to enhance acceptability and uptake of evidence-based care. Our aim was to explore perceptions of drug use, PWUD and approaches to harm reduction and treatment in an urban, predominantly Black neighborhood heavily impacted by overdose.

**Methods:**

We conducted one-on-one, semi-structured interviews with a purposive sample of participants living or working in West and Southwest Philadelphia, focusing on factors influencing uptake of substance use services. Interviews were recorded, transcribed, and analyzed using thematic analysis.

**Results:**

We completed 19 interviews. Mean participant age was 46, 79% of the sample were women; 83% were Black/AA. Half of participants worked with PWUD in health or social service roles (50%), and the majority had lived experience of substance use personally or with a close friend or family member (83%). Participants reported several factors of attitudes toward substance use, PWUD, and addiction care in the community. First, participants frequently referenced the legacy of the punitive drug policies regarding current community apprehension about substance use services. Participants reported a high degree of community stigma towards PWUD, as well as a view of harm reduction as an endorsement of drug use rather than a public health effort. Stigma also shaped cultural norms, limiting acceptability of care-seeking among PWUD. In addition, participants highlighted the toll of systemic racism, noting that it contributed to generational trauma, substance use, and overall vulnerability to addiction and overdose. Finally, participants emphasized the importance of community-driven initiatives, culturally appropriate services, and expanded outreach to actively address and dismantle the structural inequities.

**Conclusion:**

Overall, participants from West and Southwest Philadelphia described how the combined impact of the war on drugs, systemic racism, and medical system mistrust has shaped the experiences of Black PWUD and their communities. Participants highlighted the need for equitable, culturally responsive public health policies that safeguard the provision of harm reduction services for Black PWUD.

## Background

Premature death due to opioid use is a growing public health burden in the United States and an important equity issue [1]. Recent national data has shown that the overdose death rate among Black individuals is surpassing that of Non-Hispanic White individuals [2, 3]. In Philadelphia—which has the highest age-adjusted fatal overdose rate among large U.S. cities- unintentional overdose deaths were the highest among Black individuals [4]. Additionally, between 2019 and 2021, opioid and other stimulant-involved deaths rose at a much higher rate among Black individuals, and the overdose rate for the majority Black communities in West and Southwest Philadelphia increased over 30% following the COVID-19 pandemic [4, 5].

Disproportionate increases of overdose deaths in Black communities are related in part to inequities in quality and access to care for Black individuals overall as well as inequities in access to addiction treatment and harm reduction services [6–8]. Black patients report more medical mistrust and racial discrimination from healthcare workers, which has been linked to poor health outcomes across many healthcare contexts, including substance use disorder (SUD) care [9]. Recent changes in the drug supply, including contamination of opioid and non-opioid substances with fentanyl and other analogues, may also be a contributor to these trends [10, 11]. Therefore, it is critical to reach Black communities with harm reduction strategies to reduce the toll of the overdose crisis.

Despite increases in overdose deaths, there is limited research on effective strategies to increase uptake of treatment and harm reduction services in Black communities [12]. Our aims were to (1) explore community stakeholder perspectives on the barriers and facilitators to the uptake of substance use treatment and harm reduction interventions in West and Southwest Philadelphia and (2) solicit suggestions to improve delivery of these services. Our goal was to use these insights to inform best practices in building sustainable, community-driven partnerships to address the overdose crisis in this community.

## Methods

Our study included semi-structured, one-on-one interviews of community stakeholders who live or work in West and Southwest Philadelphia. The current study followed the Consolidated Criteria for Reporting Qualitative Research and was approved by the University of Pennsylvania Institutional Review Board [13].

### Study setting

This study was conducted with community members from West and Southwest Philadelphia, a predominantly black community with a long history of activism and civic engagement. Compared to Philadelphia as a whole, West Philadelphia residents experience a 10-year lower life expectancy (e.g. 67.1 years vs 78.3 years) with nearly a third of households living in poverty [14]. This community houses two prominent academic-healthcare institutions—The University of Pennsylvania and Drexel University. Formerly referred to as “The Black Bottom”, this predominantly Black neighborhood in West Philadelphia once housed between 5000 and 10,000 residents who were displaced by the City of Philadelphia in partnership with The University of Pennsylvania and Drexel University under the guise of “urban renewal” in the 1960s [15]. Like many other communities across the US, West and Southwest Philadelphia has been severely impacted by the War on Drugs. With this work, our hope is to amplify the experiences and perspectives of community leaders and create space for them to lead us towards an equitable future.

### Study participants and recruitment

We recruited adult (≥ 18 years old) community members who either lived or worked in West and Southwest Philadelphia for one-on-one, semi-structured interviews. We used purposive sampling to recruit participants from a variety of sectors, including community residents, individuals who provide services to PWUD, those with lived experience with substance use and recovery, and other community leaders such as block captains.

### Interview content

Interviews focused on the impact of substance use on the West and Southwest Philadelphia community. Domains included personal and community perceptions of substance use and PWUD, experiences accessing substance use services in West and Southwest Philadelphia, relationships between their community and the surrounding healthcare institutions, and factors impacting the uptake of harm reduction and treatment services. To gauge service acceptability, each participant was asked to describe their perspectives on harm reduction and the types of harm reduction and treatment services that would be acceptable in their community. Finally, we explored participant recommendations to build rapport with academic-healthcare institutions in West Philadelphia. The authors collaborated in the design of all research materials and consulted the senior author and other members of the study team with lived experience to create materials sensitive to the complexities of the experiences of our participants.

### Recruitment

Participants were recruited via community outreach, flyers, and email. We identified a purposive sample of community stakeholders to gauge interest in study participation and distributed recruitment materials at community outreach events. For all participants who were invited or volunteered to participate, we sent an initial brief screening survey to ensure they met inclusion criteria. Participants were then contacted by trained research assistants to confirm interest in study participation and obtain informed consent. Interviews were conducted both virtually and in-person by members of the research team (JB, MHD, KG) and lasted 45–60 min. At the conclusion of each interview, we recorded demographic information and compensated with a $50 gift card. All participants were also invited to remain engaged in work moving forward as a member of an ongoing Community Advisory Board (CAB).

### Authors’ positionality and values

This study was intentionally designed to build solidarity and trust with residents of West and Southwest Philadelphia, communities disproportionately affected by overdose and by historical and ongoing structural harms, including those perpetuated by academic and medical institutions. Our research team includes members with lived experience, deep ties to Philadelphia, and long-standing engagement with community-based harm reduction and advocacy efforts, which informed the study’s conceptualization and implementation. Our approach was grounded in an explicit acknowledgment of institutional histories that have contributed to community mistrust and a commitment to centering experiential knowledge as a critical form of expertise. Guided by values of equity, accountability, and reciprocity, we sought to interview trusted community messengers and intentionally positioned community leaders not only as participants, but as collaborators who guided programmatic priorities and recommendations as an ongoing deliverable of this work. To that end, all interested study participants were invited to participate as standing members of our team’s CAB. This reflexive, community-driven orientation informed all stages of the research process and reflects our commitment to scholarship that elevates the voices of historically marginalized communities, addresses structural inequities, and supports sustainable, trust-based collaboration.

### Analysis

Interviews were audio-recorded and transcribed using a professional service. Research assistants (JB, KG and MHD) reviewed each transcript for accuracy prior to discussion to develop the initial codebook. We used a hybrid deductive-induction approach to capture a-priori themes as well as emergent themes that arose in interviews. Using the mutually developed codebook, KM and JB double coded 10 randomly selected transcripts to establish inter-rater reliability (kappa = 0.7) using NVivo software (version 12.0, QSR International). Any discrepancies were resolved via discussion, and remaining transcripts were coded by a single research assistant. We then used thematic analysis to identify key themes through discussion with the research team [16].

## Results

We completed 19 interviews. Participants’ mean age was 46, 78% were women; 83% identified as Black or African American (Table 1). Most participants either worked with PWUD in health or social service roles (50%) and/or had lived experience personally or with a close friend/family member (83%).

**Table 1:** Participant characteristics

Characteristic, *n* (%)	Study group (*n* = 19)
*Age*	
Mean	46
*Gender*	
Female	15 (79%)
Male	4 (21%)
*Ethnicity*	
Hispanic or Latino	0
Race*	
White	3 (21%)
Black	16 (84%)
*Educational attainment*	
Did not graduate high school	2 (10%)
High school diploma/GED	4 (21%)
Some college	2 (10%)
Associate degree	1 (5%)
Bachelor’s degree	4 (21%)
Masters or higher	6 (32%)
*Employment status*	
Full-time	15 (79%)
Part-time	1 (5%)
Unemployed	3 (16%)
*Do you live in west/southwest Philadelphia?*	
Yes	14 (74%)
No	5 (26%)
*Do you work in west/southwest Philadelphia?*	
Yes	14 (74%)
No	5 (26%)
*Do you work with people who use drugs (PWUD)?*	
Yes	10 (53%)
No	9 (47%)
*Have you or a loved one been affected by drug use?*	
Yes	16 (84%)
No	3 (16%)

In the following section, we present results related to community perspectives in two key areas: (1) The influence of cultural and historical context on the uptake of harm reduction and SUD treatment initiatives, and (2) recommendations for improving relationships, as well as service delivery, between the community and healthcare institutions.

### Legacy of the war on drugs and the criminalization of substance use

Acceptability of harm reduction and treatment services was shaped by several historical and cultural factors. Overwhelmingly, participants noted the destructive impact of the War on Drugs on predominantly Black neighborhoods, including West and Southwest Philadelphia. Participants described lasting impacts, including the destabilization of families and communities through mass incarceration, deteriorating neighborhoods, and fewer opportunities for advancement. One shared:

*“Families are being broken. Children are being left alone, not cared for. Grandparents are being responsible for the care of children. More and more people are dying. More and more people are being found unresponsive on the street … Crime has increased significantly. Violence has increased significantly. {#1}”*

Although some participants agreed that harm reduction initiatives could benefit their community, many felt that uptake was influenced by prior racialized approaches to substance use and fear among PWUD that engagement in services would make them targets for law enforcement. One participant stated:

*“A lot of times people believe that getting help is calling the police, and that’s probably the worst thing you could possibly do in a situation when you’re trying to help somebody with drug usage … they don’t wanna see a police officer come to their house … because they think they’re gonna get in trouble and go to jail. {#19}”*

This fear was driven by real experiences with inappropriate, stigmatizing, or violent responses towards Black PWUD who needed help. One participant shared an experience witnessing a police interaction with a person who used drugs as follows:

*“You don’t see any EMTs come out. I watched a guy sit back and froth at the mouth from taking some bad stuff that he had the other day. The cop said, get your ass up. And the cop is hitting with the billy club and dragged him over to a paddy wagon. I’m pretty sure that guy’s dead. And the reason I say that because nobody ever saw him again.” {#4}*

Participants also noted significant differences in the historical responses towards Black PWUD were significantly different than current societal responses to White PWUD. One shared:

“*I think it’s interesting that now that substance abuse is affecting White people, now they want to have needle exchange programs …. But with the crack epidemic, all the Black people that were impacted by it went to jail or prison. Personally, I think it’s contradictory and I wonder why now all of the sudden that White people are majorly impacted by why it’s a national issue.” {#11}”*

In summary, participants expressed that the historical and current criminalization of substance use in Black communities has lasting effects on the acceptability of harm reduction and SUD treatment initiatives in West and Southwest Philadelphia.

### Impacts of inequitable resource distribution, community disinvestment, and structural racism

Participants observed clear disparities in the distribution of substance use resources and services along neighborhood—and often racial—lines. Participants perceived that much of Philadelphia’s attention and funding to address the overdose crisis went primarily to a single, historically predominantly white neighborhood—Kensington—and that local needs in West and Southwest Philadelphia were neglected. One participant who worked as a service provider shared:

*“While I was [working in West Philadelphia], I would have patients that I would tell them, you have to go to Kensington to get this service or this treatment. And that is the worst feeling for so many things, it’s like safe injection, clean supply, fresh works, all of this shit I have to always tell patients like, I know you’re trying not to use and I know you’re trying not to be out there, but the only place you can go to have these services is [Kensington].”{#17}*

In addition to the inequitable resource distribution, the consensus was that locally available substance use care was lower quality, difficult to access, or limited to emergency services. Local providers often had long wait times, staff shortages, and limited options for care modalities. One participant described their experience accessing a local mental health Crisis Response Center (CRC) as follows:

“*I’ve called CRC personally for people that have had trouble … I’ve had some complaints in the past where patients have tried to call the CRC and they don’t get through, the phone lines are jammed, they get the runaround. So that is a major barrier to care, for sure, in my experience.” {#9}*

Negative experiences with medical systems also created a sense of distrust in the community and apprehension about seeking care, and this distrust built over time. One participant shared:

*“There are people in the West Philadelphia community who will not go to certain doctors or certain hospitals or health systems because*—*not because they were treated poorly, but because their family members were. … [People] don’t want to have a negative experience when they’re in pain, when they are needing help. And people remember. The community’s memory is timeless.” {#3}*

In addition, participants described cumulative impacts of disinvestment in the community, poor access to healthcare and services, and criminalization of substance use and other impacts of structural racism as leading to collective trauma, which in turn, became a driver of substance use. One participant captured this point in the following way:

*“There’s a lot of generational trauma and things that are passed down. If you’re experiencing so much pain that you feel like you don’t have a way out and you have no one to go to and no other answer, the numbing through or at least self-soothing through substances is obviously a sign of a broken system. So, we shouldn’t be criminalizing the victims of the system. We should be fixing the system, so that people get what they need. {#5}”*

Finally, participants saw gaps in responses from institutions serving their community in addressing substance use challenges. As their community struggled to cope with shifting trends in the illicit drug supply with limited resources, they felt that local health care institutions were not addressing these challenges in a way that was responsive to current and historical challenges. One shared:

*“Unfortunately, I think for the long time everyone’s been doing a poor job of addressing [substance use] in West Philly ... I don’t know if there’s an easy answer to stop that problem. But I don’t think any of the medical centers have been really focused on that issue. It’s been one of those hush hush problems, bring them to the hospital, let them be seen in the ER, send them on their way, then we get them again and we just repeat this cycle over and over until unfortunately that one time we go, we find them, and they’re now deceased. {#19}”*

The consensus was that structural racism has lasting effects that contributes to distrust of the medical system and perpetuates the disconnect between healthcare institutions and the West Philadelphia community. Overall, participants suggested the lack of access to quality care for Black PWUD, failure to provide resources to adequately address the increase in overdose rates in West and Southwest Philadelphia, and generational trauma perpetuated inequities in outcomes.

### Participant recommendations

Community members made several recommendations to health care systems for improving services for Black PWUD and developing partnerships with the West and Southwest Philadelphia community. Most participants suggested that surrounding healthcare institutions should focus their efforts on building trust with the community through outreach, physical presence, relationship-building with community stakeholders, and advocacy***.*** Part of this process involved acknowledging past and present harms and encouraging accountability within leadership:

***“****We can’t change the things that we don’t accept, so just accepting, okay, ‘Here is our legacy as an organization. Here are the things that we’ve done in the past. We have gentrified neighborhoods, displaced people, harmed people, stolen land, just generally. So, what do we do with that now, right?’ And … not just doing these things for notoriety or PR stunts, but also just trying to enmesh with and just genuinely respect human life in our community.” ****{6}***

Other advice focused on increased community investment and an emphasis on meaningful partnership between institutions and the community. One participant advised that funding from the organizations should:

“*begin to really circulate in that community. Oftentimes, groups and educational facilities and great hospitals, they come into communities, and they don’t put anything into the community. There has to be some sort, some level of activities, programming, beautification of the community from the big organizations to say, hey, we wanna be part of this community too, and this is what we’re willing to do to be a part of that community.”**** {#1}***

Participants also stressed the importance of establishing partnerships with their community not only for the purpose of research, but also to consult with the community when planning programs and services. One shared:

“*Another thing might be doing more things like [community advisory boards], for not only research but even doing that if we’re gonna build a new clinic, or even in hospitals now … What would make people feel more safe and actually willing to go there? Because I know also a lot of folks have been discriminated in the health system before … and therefore, don’t go get the care that they need.” ****{#2}***

Finally, participants recommended training institutional staff and medical personnel on provision of culturally competent, non-stigmatizing care for Black PWUD, incorporating an anti-racism framework, and diversifying hiring practices to better reflect the surrounding community. While fostering cultural competence through training was viewed as important, several participants felt a necessary step to show commitment to anti-racism and equity was to give back to the community in the form of (monetary?) reparations. One participant advised:

**“***We can take all the antiracism trainings that we want, right, but if we’re still not paying reparations to the community, we fail [to equitably address the harms we’ve done], so it’s kind of like living as an institution those values out. And I think that’s something that a lot of institutions are questioning, what does that actually look like?” ****{#6}***

Participant recommendations were a call to action for healthcare leadership to focus on relationship building to encourage healing and rapport between West and Southwest Philadelphians and the surrounding healthcare institutions. While these recommendations were not specific to SUD treatment or PWUD more broadly, participants identified these challenges as barriers to relationship building between racialized groups and healthcare systems. Addressing these issues are essential to developing longstanding, sustainable partnerships and ultimately SUD treatment services that are both culturally appropriate and relevant to the needs of BIPOC communities.

## Discussion

Through interviews with West and Southwest Philadelphia community stakeholders, we explored community member perceptions of substance use and PWUD, uptake of treatment and harm reduction services, and ways to improve future engagement and partnership building with surrounding healthcare institutions. Our results indicate that the legacies of criminalization of drug use and structural racism not only impact community life but perpetuate inequities that stall the uptake of harm reduction initiatives and care-seeking. These findings can inform strategies to improving acceptability of services for Black communities across the U.S. experiencing similar trends in several key ways.

First, our results attest to the lasting impact of the War on Drugs on Black communities and the influence this has on acceptability of substance use-related services today. The criminalization of Black PWUD and resulting mass incarceration destabilized Black communities, with lasting impacts today [17]. The resulting neighborhood characteristics vastly contributes to poor health outcomes as communities lack access to care for their substance use or recovery-based support [18, 19]. Black PWUD continue to face higher rates of incarceration for substance-related crimes and are less likely to be offered medications for opioid use disorder in comparison to their White counterparts [20–22]. Overwhelmingly, participants in our study described apprehension about harm reduction approaches in predominantly Black communities as being a direct result of decades of criminalized approaches to drug use. Due to limited support and access to harm reduction services, Black PWUD often perceive these practices less favorably, viewing them as potential endorsements of substance use that could invite police presence. Existing literature highlights the present-day challenges Black communities face accessing and accepting harm reduction services, in part due to these factors [23]. These barriers reflect broader systemic issues that restrict opportunities for Black PWUD to engage in evidence-based, stigma-free care.

Second, our findings provide important insights into the relationship between the systemic drivers of substance use and overdose. Participants noted community perceptions that treatment and harm reduction messaging disproportionally support White PWUD, compounded by lower availability of these services in their community, therefore limiting uptake and access. While our study was limited to the experiences of Black West and Southwest Philadelphians, recent literature highlights the intersections of systemic racism and the lack of support available to Black PWUD as a continuation of neglect that Black communities have grappled with throughout time. Recent literature highlights the policies and practices (e.g., redlining, gentrification, discriminatory lending, etc.) and the impact of repeatedly disadvantaging marginalized populations on the development of substance use disorders [24, 25]. Prior work has demonstrated a clear correlation between systemic inequities and overdose risk in Black and Brown neighborhoods. Published literature highlights that lack of access to quality healthcare, inequitable resource distribution, and other upstream factors coupled with interventions that aim to improve SUD disparities without addressing the underlying social inequities often fail to achieve their aim, resulting in worsening outcomes for racially minoritized groups [26–28]. Participants overwhelmingly highlighted the lack of options available for care in historically black neighborhoods in comparison to other neighborhoods in Philadelphia, like Kensington, which has multiple options for low-barrier services to support PWUD. Our participants suggested that differing public health responses perpetuated disparities, with West and Southwest Philadelphia receiving fewer resources than other areas of the city. Participants also suggested several strategies for public health and health care organizations, including increased funding to support expansion of available treatment options and education and awareness for first responders and lay persons to support their community. They also advised engaging community leaders in the design, implementation and evaluation of community programs to foster relationship and build trust, to increase acceptability of harm reduction services and ensure cultural sensitivity. In addition to centering the community when designing SUD services, numerous upstream, policy-based solutions exist—like decriminalizing drug possession and advocating for policy reform to support communities impacted by the War on Drugs.

Third, our results highlight the cumulative effects of structural racism in perpetuating trauma and repeatedly disadvantaging minoritized communities. The emotional, physical and psychological impact of racism and disenfranchisement are well documented, and the resulting stressors contribute to collective trauma experienced by Black, urban communities [29–31]. Further, discriminatory policies that concentrate funding into predominantly White neighborhoods and neglect the needs of minority communities further isolate Black PWUD and their communities, resulting in fewer opportunities for advancement and equitable access to care [32, 33]. Participants in our study also stressed the role of gentrification and displacement as factors that impede relationship building and damage rapport between West and Southwest Philadelphia and surrounding healthcare institutions. This makes it difficult to build trust, which is essential to improve care engagement, both broadly and more specifically for substance use care [34, 35]. Research suggests that systemic inequities, such as gentrification, redlining and other racialized forms of discrimination further contribute to neighborhood disinvestment, poor neighborhood conditions and concentrated poverty, thereby limiting opportunities for homeownership and other forms of generational wealth building [36, 37]. The interplay of these broader structural factors, combined with a rapidly changing drug supply, may play a role in the vulnerability and increasing deaths among Black PWUD.

Finally, participants highlight the importance of representation, inclusion, and reparations to build trust and foster culturally sensitive, safer spaces for Black PWUD. The benefits of engaging with healthcare personnel one identifies with culturally are well documented elsewhere [38–40]. Our findings stress the urgency of recruiting personnel who represent the community and connecting with community members through street outreach. Participants stressed the importance of increasing access to SUD care, recruiting culturally representative personnel and increasing accessibility of harm reduction resources and supplies. Our results overwhelmingly suggest that our primary aim should be to address the systemic drivers of disparities, not to “fix” individuals or their health behaviors, which is consistent with a wealth of literature that solidifies the efficacy of upstream public health interventions to address the opioid epidemic [41, 42]. Participant recommendations also centered the importance of empathy and connection to build relationships and shift towards a more inclusive, community-driven approach to care and harm reduction services. Previous studies have revealed that Black PWUD are most often stigmatized and receive a less empathetic approach when encountering the healthcare system [29, 31, 43]. In this study, stigma is operationalized as a multi-level, intersecting phenomenon that operates across interpersonal, institutional, and structural domains, shaping how Black PWUD are perceived, treated, and resourced within healthcare and social service systems. Participants’ narratives illustrate how racialized drug stigma, medical mistrust, and criminalization converge to produce compounded barriers to care, diminished clinical empathy, and heightened overdose vulnerability. Consistent with prior literature, these findings suggest that stigma cannot be understood as a singular or individual-level phenomenon, but rather as a result of fundamental structural forces that perpetuate inequities in care quality, access and health outcomes for marginalized communities [44–47]. Overall, our findings emphasize that building trust and creating space for the community at all levels of SUD program planning and implementation are essential to genuinely serve and empower Black PWUD.

## Limitations

Our study has several limitations. While our findings represent the views of a diverse group of individuals in West and Southwest Philadelphia, our study population skewed towards highly educated, female, and employed. Although we included people who worked professionally with PWUD and/or with personal lived or family experiences with substance use, we did not include any people with current living experience with substance use who may have different perspectives. Finally, since our focus was participants who either live or work in West and Southwest Philadelphia, our findings may not be generalizable to all Black communities impacted by substance use.

## Conclusions

In conclusion, the combined impact of the war on drugs, systemic racism, and medical system mistrust has profoundly shaped the experiences of Black PWUD and their communities. Participants shared how decades of punitive drug policies and racially biased enforcement have deepened existing social and economic inequalities, isolating Black PWUD from supportive health and social services. Addressing these harms requires a shift to a public health model that prioritizes culturally competent care, equitable access to resources, and community-centered harm reduction services.

## Data Availability

Data sharing is not applicable to this article as no datasets were generated or analyzed during the current study.

## Abbreviations

PWUD: People who use drugs
SUD: Substance use disorder
CAB: Community advisory board

## References

[1] Gomes T, Tadrous M, Mamdani MM, Paterson JM, Juurlink DN. The burden of opioid-related mortality in the United States. JAMA Netw Open. 2018;1(2):e180217. 10.1001/jamanetworkopen.2018.0217. 30646062 10.1001/jamanetworkopen.2018.0217 PMC6324425

[2] Cano M, Sparks CS. Drug overdose mortality by race/ethnicity across US-born and immigrant populations. Drug Alcohol Depend. 2022;232:109309. 10.1016/j.drugalcdep.2022.109309. 35077954 10.1016/j.drugalcdep.2022.109309

[3] D’Orsogna MR, Böttcher L, Chou T. Fentanyl-driven acceleration of racial, gender and geographical disparities in drug overdose deaths in the United States. PLoS Glob Public Health. 2023;3(3):e0000769. 10.1371/journal.pgph.0000769. 36962959 10.1371/journal.pgph.0000769 PMC10032521

[4] Philadelphia Department of Public Health. Unintentional Drug Overdose Fatalities in Philadelphia, 2022; 2023. https://www.phila.gov/media/20231002090544/CHARTv8e3.pdf.

[5] Harris RA. Drug overdose deaths among Non-Hispanic Black men in the U.S.: age-specific projections through 2025. AJPM Focus. 2022;2(1):100063. 10.1016/j.focus.2022.100063. 37377540 10.1016/j.focus.2022.100063 PMC10299749

[6] Mays VM, Jones A, Delany-Brumsey A, Coles C, Cochran SD. Perceived discrimination in healthcare and mental health/substance abuse treatment among Blacks, Latinos, and Whites. Med Care. 2017;55(2):173–81. 10.1097/MLR.0000000000000638. 27753743 10.1097/MLR.0000000000000638 PMC5233585

[7] Parlier-Ahmad AB, Pugh M, Martin CE. Treatment outcomes among Black adults receiving medication for opioid use disorder. J Racial Ethn Health Disparities. 2022;9(4):1557–67. 10.1007/s40615-021-01095-4. 34254271 10.1007/s40615-021-01095-4 PMC8274965

[8] Hulsey JN. Toward improved addiction treatment quality and access for Black patients. Am J Public Health. 2022;112(S1):S21–3. 10.2105/AJPH.2021.306664. 35143277 10.2105/AJPH.2021.306664 PMC8842210

[9] Hall OT, Jordan A, Teater J, et al. Experiences of racial discrimination in the medical setting and associations with medical mistrust and expectations of care among Black patients seeking addiction treatment. J Subst Abuse Treat. 2022;133:108551. 10.1016/j.jsat.2021.108551. 34244014 10.1016/j.jsat.2021.108551

[10] Ciccarone D. The triple wave epidemic: supply and demand drivers of the US opioid overdose crisis. Int J Drug Policy. 2019;71:183–8. 10.1016/j.drugpo.2019.01.010. 30718120 10.1016/j.drugpo.2019.01.010 PMC6675668

[11] Ivsins A, Boyd J, Beletsky L, McNeil R. Tackling the overdose crisis: the role of safe supply. Int J Drug Policy. 2020;80:102769. 10.1016/j.drugpo.2020.102769. 32446183 10.1016/j.drugpo.2020.102769 PMC7252037

[12] Jackson DS, Nguemeni Tiako MJ, Jordan A. Disparities in addiction treatment: learning from the past to forge an equitable future. Med Clin North Am. 2022;106(1):29–41. 10.1016/j.mcna.2021.08.008. 34823733 10.1016/j.mcna.2021.08.008

[13] Tong A, Sainsbury P, Craig J. Consolidated criteria for reporting qualitative research (COREQ): a 32-item checklist for interviews and focus groups. Int J Qual Health Care. 2007;19(6):349–57. 10.1093/intqhc/mzm042. 17872937 10.1093/intqhc/mzm042

[14] *Health of the City: A Report on Community Health in Philadelphia*. Philadelphia Department of Public Health; 2023. https://philadelphiapublichealth.shinyapps.io/health-of-the-city

[15] Wolf-Powers L. University City: history, race, and community in the era of the Innovation District. University of Pennsylvania Press; 2022.

[16] Pope C, Mays N. Qualitative research in health care. 4th ed.; 2006.

[17] Alexander M. The new jim crow. Penguin Books; 2019.

[18] Jackson DS, Tiako MJN, Jordan A. Disparities in addiction treatment: Learning from the past to forge an equitable future. Med Clin (Barc). 2022;106(1):29–41. 10.1016/j.mcna.2021.08.008. 10.1016/j.mcna.2021.08.008 34823733

[19] James K, Jordan A. The Opioid Crisis in Black communities. J Law Med Ethics. 2018;46(2):404–21. 10.1177/1073110518782949. 30146996 10.1177/1073110518782949

[20] Cooper HLF, Cloud DH, Fanucchi LC, Lofwall M, Young AM. Dismantling war on drugs policies in COVID-19’s aftermath. Am J Public Health. 2022;112(S1):S24–7. 10.2105/AJPH.2021.306680. 35143266 10.2105/AJPH.2021.306680 PMC8842201

[21] Hollander MAG, Chang CCH, Douaihy AB, Hulsey E, Donohue JM. Racial inequity in medication treatment for opioid use disorder: exploring potential facilitators and barriers to use. Drug Alcohol Depend. 2021;227:108927. 10.1016/j.drugalcdep.2021.108927. 34358766 10.1016/j.drugalcdep.2021.108927 PMC8464525

[22] Lagisetty PA, Ross R, Bohnert A, Clay M, Maust DT. Buprenorphine treatment divide by race/ethnicity and payment. JAMA Psychiat. 2019;76(9):979–81. 10.1001/jamapsychiatry.2019.0876. 10.1001/jamapsychiatry.2019.0876 PMC6506898 31066881

[23] Cohen A, Vakharia SP, Netherland J, Frederique K. How the War on Drugs impacts social determinants of health beyond the criminal legal system. Ann Med. 2022;54(1):2024. 10.1080/07853890.2022.2100926. 35852299 10.1080/07853890.2022.2100926 PMC9302017

[24] Amaro H, Sanchez M, Bautista T, Cox R. Social vulnerabilities for substance use: stressors, socially toxic environments, and discrimination and racism. Neuropharmacology. 2021;188:108518. 10.1016/j.neuropharm.2021.108518. 33716076 10.1016/j.neuropharm.2021.108518 PMC8126433

[25] Ebrahimi CT, Polanco-Roman L, Saraiya TC, Bauer AG, Hien D. Historical trauma and polysubstance use in Black young adults: the role of contemporary racism. Psychol Trauma. 2024;16(6):922–9. 10.1037/tra0001652. 38300572 10.1037/tra0001652 PMC11291707

[26] Friedman JR, Nguemeni Tiako MJ, Hansen H. Understanding and addressing widening racial inequalities in drug overdose. Am J Psychiatry. 2024;181(5):381–90. 10.1176/appi.ajp.20230917. 38706336 10.1176/appi.ajp.20230917 PMC11076008

[27] Jegede O, Bellamy C, Jordan A. Systemic racism as a determinant of health inequities for people with substance use disorder. JAMA Psychiat. 2024;81(3):225–6. 10.1001/jamapsychiatry.2023.4958. 10.1001/jamapsychiatry.2023.4958 38231489

[28] Lopez AM, Thomann M, Dhatt Z, et al. Understanding racial inequities in the implementation of harm reduction initiatives. Am J Public Health. 2022;112(S2):S173–81. 10.2105/AJPH.2022.306767. 35349311 10.2105/AJPH.2022.306767 PMC8965181

[29] Bailey ZD, Feldman JM, Bassett MT. How structural racism works—racist policies as a root cause of U.S. racial health inequities. N Engl J Med. 2021;384(8):768–73. 10.1056/NEJMms2025396. 33326717 10.1056/NEJMms2025396 PMC11393777

[30] Riley AR. Neighborhood disadvantage, residential segregation, and beyond—lessons for studying structural racism and health. J Racial Ethn Health Disparities. 2018;5(2):357–65. 10.1007/s40615-017-0378-5. 28573643 10.1007/s40615-017-0378-5

[31] Stopforth S, Kapadia D, Nazroo J, Bécares L. The enduring effects of racism on health: understanding direct and indirect effects over time. SSM Popul Health. 2022;19:101217. 10.1016/j.ssmph.2022.101217. 36091297 10.1016/j.ssmph.2022.101217 PMC9450139

[32] Egede LE, Walker RJ, Williams JS. Addressing structural inequalities, structural racism, and social determinants of health: a vision for the future. J Gen Intern Med. 2024;39(3):487–91. 10.1007/s11606-023-08426-7. 37740168 10.1007/s11606-023-08426-7 PMC10897090

[33] Lynch EE, Malcoe LH, Laurent SE, Richardson J, Mitchell BC, Meier HCS. The legacy of structural racism: Associations between historic redlining, current mortgage lending, and health. SSM Popul Health. 2021;14:100793. 10.1016/j.ssmph.2021.100793. 33997243 10.1016/j.ssmph.2021.100793 PMC8099638

[34] Cockroft JD, Adams SM, Bonnet K, Matlock D, McMillan J, Schlundt D. “A Scarlet Letter”: stigma and other factors affecting trust in the health care system for women seeking substance abuse treatment in a community setting. Subst Abuse. 2019;40(2):170–7. 10.1080/08897077.2018.1544184. 10.1080/08897077.2018.1544184 30759047

[35] Versey HS. Gentrification, health, and intermediate pathways: how distinct inequality mechanisms impact health disparities. Hous Policy Debate. 2023;33(1):6–29. 10.1080/10511482.2022.2123249.

[36] Egede LE, Walker RJ, Campbell JA, Linde S, Hawks LC, Burgess KM. Modern day consequences of historic redlining: finding a path forward. J Gen Intern Med. 2023;38(6):1534–7. 10.1007/s11606-023-08051-4. 36746831 10.1007/s11606-023-08051-4 PMC9901820

[37] South E, Venkataramani A, Dalembert G. Building black wealth—the role of health systems in closing the gap. N Engl J Med. 2022;387(9):844–9. 10.1056/NEJMms2209521. 36053512 10.1056/NEJMms2209521

[38] Jetty A, Jabbarpour Y, Pollack J, Huerto R, Woo S, Petterson S. Patient-physician racial concordance associated with improved healthcare use and lower healthcare expenditures in minority populations. J Racial Ethn Health Disparities. 2022;9(1):68–81. 10.1007/s40615-020-00930-4. 33403653 10.1007/s40615-020-00930-4

[39] Ma A, Sanchez A, Ma M. The impact of patient-provider race/ethnicity concordance on provider visits: updated evidence from the Medical Expenditure Panel Survey. J Racial Ethn Health Disparities. 2019;6(5):1011–20. 10.1007/s40615-019-00602-y. 31236800 10.1007/s40615-019-00602-y

[40] Ma A, Sanchez A, Ma M. Racial disparities in health care utilization, the Affordable Care Act and racial concordance preference. Int J Health Econ Manag. 2022;22(1):91–110. 10.1007/s10754-021-09311-8. 34427837 10.1007/s10754-021-09311-8

[41] Salmond S, Allread V. A population health approach to America’s opioid epidemic. Orthop Nurs. 2019;38(2):95–108. 10.1097/NOR.0000000000000521. 30768537 10.1097/NOR.0000000000000521 PMC6519712

[42] Samuels EA, Doran KM. Moving upstream: a social emergency medicine approach to opioid use disorder. Ann Emerg Med. 2022;79(2):168–71. 10.1016/j.annemergmed.2021.08.012. 34756453 10.1016/j.annemergmed.2021.08.012

[43] Riley AR. Neighborhood disadvantage, residential segregation, and beyond-lessons for studying structural racism and health. J Racial Ethn Health Disparities. 2018;5(2):357–65. 10.1007/s40615-017-0378-5. 28573643 10.1007/s40615-017-0378-5

[44] Tsai AC, Kiang MV, Barnett ML, et al. Stigma as a fundamental hindrance to the United States opioid overdose crisis response. PLoS Med. 2019;16(11):e1002969. 10.1371/journal.pmed.1002969. 31770387 10.1371/journal.pmed.1002969 PMC6957118

[45] Latkin CA, Gicquelais RE, Clyde C, et al. Stigma and drug use settings as correlates of self-reported, non-fatal overdose among people who use drugs in Baltimore, Maryland. Int J Drug Policy. 2019;68:86–92. 10.1016/j.drugpo.2019.03.012. 31026734 10.1016/j.drugpo.2019.03.012 PMC6535351

[46] Seo DC, Alba-Lopez L, Satterfield N, Lee SH, Crabtree C, Williamson F. “There’s no real urgency when it comes to us”: critical discourse analysis of Black communities’ lived experience with opioid overdose response in Indianapolis area. Soc Sci Med. 2025;373:118039. 10.1016/j.socscimed.2025.118039. 40187070 10.1016/j.socscimed.2025.118039

[47] Hagle HN, Martin M, Winograd R, et al. Dismantling racism against Black, Indigenous, and people of color across the substance use continuum: a position statement of the association for multidisciplinary education and research in substance use and addiction. Subst Abuse. 2021;42(1):5–12. 10.1080/08897077.2020.1867288. 10.1080/08897077.2020.1867288 33465013

